# Frailty across Hip arthroplasty pathways: vulnerability domains, outcomes, and implications for care—a narrative review

**DOI:** 10.3389/fsurg.2026.1957876

**Published:** 2026-09-22

**Authors:** Feifei Li, Qin Pan, Yi Tang, Yuanwen Ye, Wentie Xu

**Affiliations:** 1 Department of Anesthesia and Surgery, The Wenzhou Third Clinical Institute Affiliated to Wenzhou Medical University, Wenzhou People’s Hospital, Wenzhou, China; 2 Department of Emergency, The Wenzhou Third Clinical Institute Affiliated to Wenzhou Medical University, Wenzhou People’s Hospital, Wenzhou, China

**Keywords:** frailty, frailty index, hip arthroplasty, postoperative outcomes, rehabilitation, sarcopenia

## Abstract

**Background:**

Frailty helps explain why older patients with similar chronological age and comorbidity burdens experience markedly different outcomes after hip arthroplasty. Interpretation is complicated because instruments capture different vulnerabilities and elective primary, revision, and fracture-related arthroplasty impose distinct stresses and intervention windows.

**Review approach:**

PubMed and Web of Science Core Collection were searched from inception through 27 July 2026, followed by a targeted PubMed search on 29 July 2026. Eligibility, staged title-and-abstract screening, an explicit supplementary-search relevance score, and selection counts were documented. Evidence was synthesized narratively by vulnerability domain, procedure context, outcome, and potential action; no meta-analysis or uniform risk-of-bias appraisal was performed.

**Main findings:**

Cumulative-deficit indices support rapid risk stratification but may overlook cognition, nutrition, mobility, and social support. Physical frailty and sarcopenia are more closely related to functional recovery, whereas multidimensional assessment can identify interacting and potentially actionable vulnerabilities. Nutritional-inflammatory indices may add modest mortality-prediction information in hip-fracture surgery, but they partly reflect acute illness and remain adjunct markers rather than validated core frailty domains. Evidence is stronger for prognostic association than for the effectiveness of frailty-guided interventions.

**Conclusions:**

A clinically useful pathway should combine low-burden screening with targeted vulnerability-domain assessment, incorporate procedure context, and repeat assessment during recovery. Frailty should guide proportional monitoring, rehabilitation, discharge planning, and shared decisions rather than serve as an isolated threshold for denying surgery. Research priorities include context-specific validation, standardized patient-centred and resource outcomes, longitudinal assessment, and trials of domain-informed care.

## Introduction

## 1 Background

Total hip arthroplasty (THA) can relieve pain and restore mobility in patients with end-stage hip disease, while THA and hemiarthroplasty remain central options for selected older adults with displaced femoral-neck fractures. As surgical candidates become older and more clinically complex, chronological age alone provides an increasingly incomplete account of perioperative vulnerability. Patients of similar age and with apparently comparable disease burdens may follow very different courses: some regain mobility and return home promptly, whereas others develop delirium or medical complications, require prolonged hospitalization or institutional rehabilitation, are readmitted, or die. Measures of frailty have therefore become attractive tools for characterizing physiological reserve and resilience (1–3).

The term hip arthroplasty, however, encompasses distinct clinical pathways. Elective primary THA offers time for optimization, shared decision-making, and prehabilitation. Revision THA adds indication-specific technical complexity, prior functional loss, and greater procedural stress. Arthroplasty after femoral-neck fracture occurs after an acute injury, when pain, inflammation, immobility, cognitive disturbance, and time pressure can obscure the prefracture baseline. A frailty score may consequently have a different meaning—and support a different intervention—in elective, revision, and emergency settings (4–6). Surgical context should therefore be treated as a potential effect modifier and essential interpretive factor rather than a minor adjustment variable.

Frailty itself is not a single construct. Throughout this review, cumulative deficits, physical reserve, nutrition, cognition, and social-functional capacity are described as vulnerability domains. The term physical frailty phenotype is reserved for the Fried construct, and no domain is presented as an empirically derived subtype. The Frailty Index (FI) and modified Frailty Index (mFI) quantify accumulated deficits; the Hospital Frailty Risk Score (HFRS) uses coded diagnoses; clinical or comprehensive geriatric approaches incorporate judgement, function, cognition, nutrition, and social circumstances; and physical frailty or sarcopenia measures focus on strength, mobility, activity, and muscle status (6–13). These instruments overlap but are not interchangeable. Instrument selection depends on the clinical setting, target outcome, available data, and decision to be informed.

Available evidence consistently associates frailty with complications, mortality, slower recovery, longer hospitalization, non-home discharge, readmission, and higher costs (2, 3, 14). Clinical implementation remains difficult because constructs and thresholds differ, elective and emergency pathways are often pooled, and most studies examine association or model discrimination rather than the effectiveness of frailty-guided care. The distinctive contribution of this review is to connect vulnerability domain x surgical context x outcome x potential action while separating elective primary, revision, and fracture-related arthroplasty. This differs from prior syntheses centred on a single instrument, one outcome, or broad mixed-joint populations. Figure 1 summarizes the resulting domain-context-outcome-action framework. Mixed THA/TKA and broader hip-fracture evidence is used only to address a defined gap and is labelled as indirect or partially direct.

**Figure 1:**
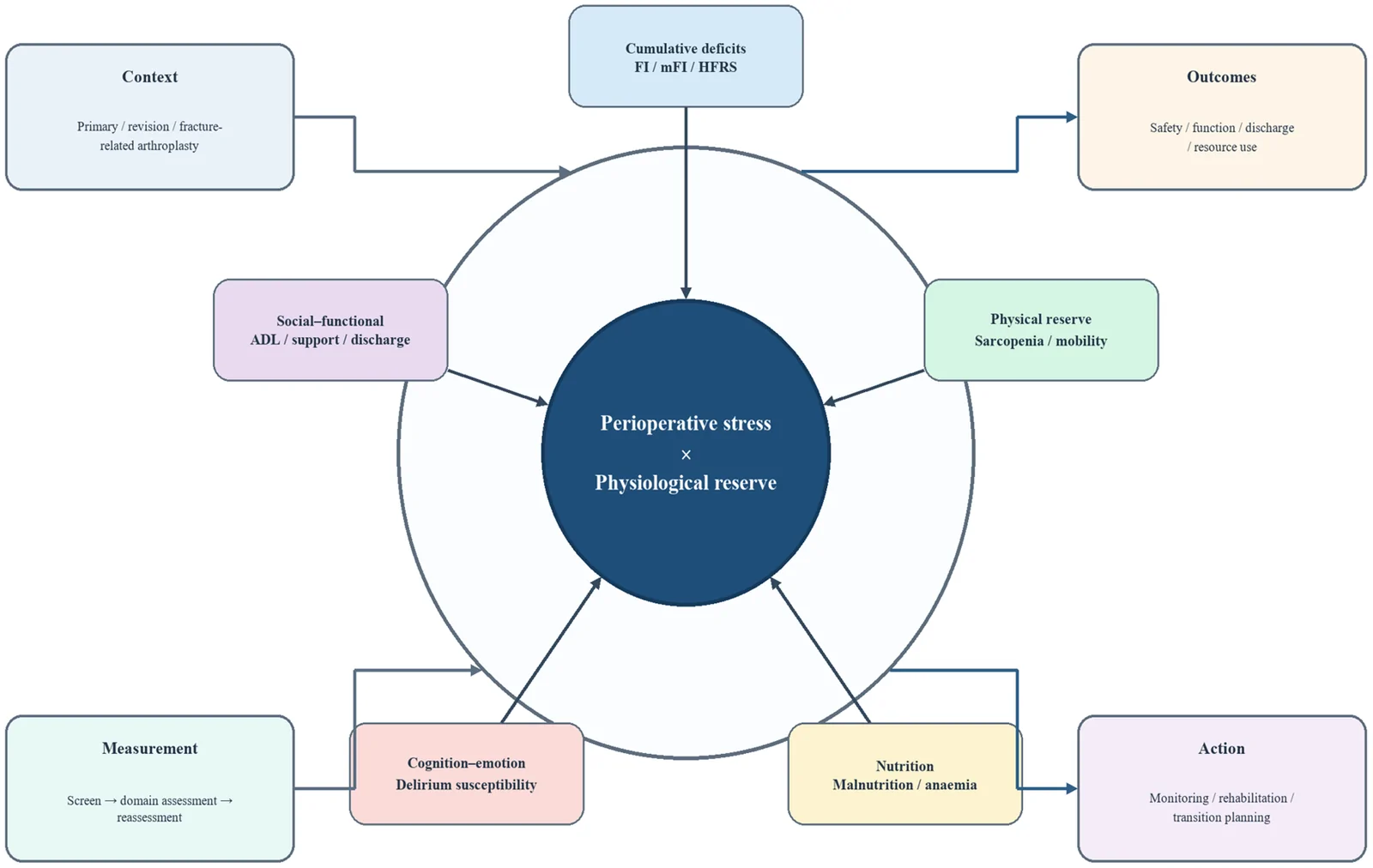
Vulnerability domain-context-outcome-action framework. The central interaction between perioperative stress and physiological reserve is surrounded by cumulative-deficit, physical, nutritional, cognitive-emotional, and social-functional domains. Arthroplasty context and measurement determine how vulnerability is interpreted; the resulting information may support outcome-specific monitoring, rehabilitation, optimization, and transitional care. Arrows denote hypothesized or evidence-supported relationships and do not establish causation or treatment effectiveness. Original figure created for this review from a multi-study synthesis (2, 5, 6, 14, 15). ADL, activities of daily living; FI, frailty index; HFRS, Hospital frailty risk score; ICU, intensive care unit; LOS, length of stay; mFI, modified frailty index; THA, total hip arthroplasty.

### 1.1 Review approach

PubMed and Web of Science Core Collection were searched from database inception through 27 July 2026. Eligible records addressed a frailty measure or a defined physical, nutritional, cognitive, or social-functional vulnerability in elective primary THA, revision THA, fracture-related THA/hemiarthroplasty, or a mixed-joint/hip-fracture population that filled a prespecified evidence gap. We excluded records without relevant vulnerability or outcome content, non-human studies, duplicates, and records that could not support a substantive statement; grey literature, registries, conference abstracts, and non-peer-reviewed reports were not used as primary evidence. The original searches yielded 235 PubMed and 233 Web of Science records (468 merged; 362 unique after deduplication). Staged title-and-abstract screening retained 111 strongly relevant records in the topic library. Forty-two were cited because they supported a traceable claim, supplied a classic instrument or consensus definition, or represented a key synthesis. A targeted PubMed search on 29 July 2026 retrieved 292 records, of which 151 overlapped with the original export and 141 were additional. An auditable relevance score assigned 6 points for a hip-arthroplasty term in the title, 5 for frailty/frail/sarcopenia in the title, 4 for a named frailty or geriatric-assessment tool in the title or abstract, 2 for a target context or outcome, and 2 for review/meta-analysis/guideline publication type. Thirty-four additional records scoring at least 9 were designated high priority for scientific review; six were incorporated into the synthesis. The reviewer-suggested Karahan study was added during revision, giving 49 cited sources. Screening was conducted for a framework-oriented narrative review and was not a PRISMA systematic-review process. Full strings, scoring rules, counts, and a narrative evidence-selection flow (Supplementary Figure 1) are provided in Supplementary File 1. No new meta-analysis or uniform risk-of-bias assessment was performed because designs, constructs, populations, and outcomes were too heterogeneous for one appraisal instrument; this limits comparative certainty.

OpenAI Codex was used solely for language editing and citation-format checking; the authors reviewed and edited the final manuscript, verified all evidence and references, and assume full responsibility for the content.

## 2 Frailty domains and measurement

### 2.1 Cumulative deficits and abbreviated indices

The deficit-accumulation model defines frailty as the proportion of health deficits present within a prespecified set. A standard FI construction procedure selects age-related health deficits spanning multiple systems, codes each deficit between zero and one, and calculates the proportion present (7). A comprehensive FI can incorporate chronic disease, symptoms, cognition, function, and geriatric assessment findings, thereby representing the breadth of vulnerability rather than a single pathway. In patients with hip fracture, an FI derived from comprehensive geriatric assessment integrated information that was less informative when its components were considered separately and was associated with mortality, length of stay, and return to the previous residence (12). Its principal limitation is dependence on the selected deficits, missing-data rules, cut-points, and timing of assessment.

The mFI family reduces this burden by using a small set of routinely available comorbidity and dependence variables. In THA databases, higher mFI-5 scores have been associated with graded increases in short- and longer-term adverse outcomes (16), and a cohort from a resource-constrained setting found the score practical for identifying patients at higher risk of complications and prolonged hospitalization (17). In patients undergoing THA or hemiarthroplasty for femoral-neck fracture, mFI-5 was more consistently associated with morbidity and readmission than with mortality (4). These findings support the mFI as an efficient risk marker, particularly when only structured variables are available.

These abbreviated tools, however, capture only part of vulnerability. Most are dominated by comorbidity and dependence and may omit cognition, nutrition, mobility quality, psychological state, and social support. In a Danish registry of 33,758 elective THA/TKA procedures, only a minority of patients who died or required intensive care crossed the study's mFI frailty threshold (1). A low abbreviated score may therefore miss risk arising from unmeasured domains or acute physiology. Local calibration is essential because coding intensity, frailty prevalence, access, and baseline function vary across health systems. Reports should specify instrument version, included deficits or domains, cut-points, assessment timing, missing-data handling, outcome definitions, calibration, and threshold consequences; discrimination alone is insufficient.

### 2.2 Physical, sarcopenic, and nutritional vulnerability

The Fried physical frailty phenotype emphasizes unintentional weight loss, exhaustion, weakness, slowness, and low physical activity (10). Sarcopenia overlaps with this phenotype but is a muscle disease for which low muscle strength indicates probable sarcopenia, low muscle quantity or quality confirms the diagnosis, and poor physical performance indicates severe sarcopenia in the EWGSOP2 consensus (11). The terms should not be used interchangeably: a patient may meet a sarcopenia definition without accumulating many medical deficits, or have a high mFI despite relatively preserved muscle performance. Physical and sarcopenic measures may be particularly relevant to rehabilitation, whereas cumulative-deficit indices are better suited to broad risk stratification (3, 6).

Direct THA evidence supports the functional relevance of sarcopenia severity. Severe preoperative sarcopenia has been associated with delayed functional recovery, non-home discharge, poorer hip function, and worse patient-reported outcomes after THA (18). A propensity-matched study of older elective THA patients similarly linked sarcopenia to poorer function at discharge and more postoperative adverse outcomes (19). Osteosarcopenia may identify patients with limited combined bone and muscle reserve, although available definitions and observational data remain insufficient for a universal clinical threshold (20). A recent systematic review and meta-analysis of total joint arthroplasty further associated sarcopenia with selected medical and implant-related outcomes, although hip-specific estimates were limited and heterogeneous (21).

Nutritional vulnerability is related to, but not reducible to, sarcopenia or frailty. Low albumin, weight loss, anaemia, inadequate intake, and inflammatory markers may reflect chronic disease, acute injury, infection, or potentially modifiable nutritional risk. Frailty and hypoalbuminaemia were only weakly correlated in elective THA, yet their coexistence identified a group with a greater short-term burden of complications and mortality than either feature alone (5). This interaction supports multidomain assessment but does not establish that correcting albumin alone will reverse risk. Similarly, resistance training with or without beta-hydroxy-beta-methylbutyrate improved selected muscle and strength measures after hip replacement without parallel between-group improvements in activities of daily living or hip-specific function (22). Improvement in one domain may occur without broader functional recovery.

Composite nutritional-inflammatory indices may add prognostic information but require particularly cautious interpretation. Karahan et al. studied 517 adults aged at least 65 years undergoing hip-fracture surgery in a multicentre retrospective cohort; the procedures were not reported as a separable arthroplasty subgroup (48). One-year mortality was 28.8%. Adding albumin, C-reactive protein, Prognostic Nutritional Index, and Geriatric Nutritional Risk Index to a clinical-frailty model increased the area under the curve from 0.666 to 0.673; adding the C-reactive protein-albumin-lymphocyte (CALLY) and Global Immuno-Nutrition Inflammation (GINI) indices increased it to 0.678. These gains were modest, information criteria worsened with model complexity, and severe multicollinearity affected frailty and nutritional variables. The study had no external validation. CALLY and GINI are therefore best regarded as adjunct markers of acute nutritional-inflammatory state and mortality risk, not established core frailty domains or evidence that nutrition-targeted treatment improves survival.

### 2.3 Cognitive and social–functional vulnerability

Cognition influences baseline assessment, pain communication, delirium susceptibility, shared decisions, and participation in rehabilitation. Preoperative frailty has been associated with early postoperative delirium after hip arthroplasty (23). In elective THA, baseline frailty or prefrailty was also associated with cognitive decline at three and twelve months, particularly when measured with a more extensive multidomain scale (24). A systematic review in primary THA/TKA linked cognitive impairment to delirium, cognitive decline, non-home discharge, and mortality, although the mixed-joint population limits hip-specific inference (25). Cognition warrants separate assessment even when comorbidity-based scores are low; conversely, cognitive impairment alone does not establish global frailty.

Social–functional vulnerability includes activities-of-daily-living dependence, mobility aids, living arrangements, caregiver availability, health literacy, deprivation, and access to rehabilitation. These factors influence discharge feasibility and recovery opportunities as well as biological risk. In Medicare beneficiaries undergoing THA/TKA, frailty was associated with greater prehabilitation use, but utilization also varied with access-related characteristics (26). Limited use may reflect constrained access as well as low need, and non-home discharge may represent appropriate supervised recovery in some settings. Patient vulnerability and health-system response must be distinguished.

The Clinical Frailty Scale (CFS), originally developed as a seven-category global clinical judgement measure, and comprehensive geriatric assessment can contextualize these domains but require judgement, personnel, and time (9). A selected cohort of adults aged at least 80 years with low CFS scores reported favourable outcomes after THA for displaced femoral-neck fracture; the retrospective design and selected population do not support a universal CFS eligibility threshold (27). HFRS occupies the opposite end of the data spectrum: it was developed from ICD-10-coded electronic hospital records to support system-level identification of older patients at risk, but is sensitive to coding and cannot explain why an individual is vulnerable or which intervention is appropriate (8, 13). Table 1 compares the constructs, data requirements, intended uses, and limitations of multidimensional, physical, bedside, and administrative approaches (6, 13, 18, 19). In a recent elective THA/TKA cohort, higher CFS categories were associated with longer hospital stay, greater inpatient-rehabilitation use, and more complications; both robust and non-robust groups nevertheless improved in patient-reported outcomes (28). Recent primary-THA data also linked the Risk Analysis Index to 30-day periprosthetic fracture, but this single-outcome database analysis requires external validation before threshold-based use (29).

**Table 1:** Frailty tools, vulnerability-domain coverage, evidence context, and validation gaps in hip arthroplasty.

Tool/domain	Construct and data	Best use	Evidence context	External validation by pathway (E/R/F)	References
FI	Multidomain cumulative deficits	Broad risk characterization	Direct THA plus indirect hip fracture	E: limited; R: NI; F: indirect only. No universal threshold	(6, 7, 12)
mFI-5/mFI-11	Comorbidity and dependence	Low-burden stratification	Direct THA; fracture arthroplasty; mixed THA/TKA	E: limited; R: mixed-joint only; F: observational, external calibration NI	(4, 16, 30)
Age-adjusted mFI	mFI plus age	Primary/revision risk prediction	Direct primary THA; mainly mixed revision THA/TKA	E: limited; R: mixed-joint only; F: NI	(31, 32)
HFRS	ICD-coded diagnoses	System screening/resource planning	Mixed primary/revision THA/TKA; indirect acute-care derivation	E/R: mixed-joint only; F: NI. Coding/actionability gaps	(8, 13)
CFS/CGA	Clinical judgement plus function/cognition	Bedside contextual assessment	Selected fracture THA; mixed TJA; broader hip fracture	E: mixed-joint only; R: NI; F: selected-cohort only	(9, 12, 27, 28)
RAI	Multidomain surgical risk	Mortality/complication stratification	Direct primary THA; periprosthetic hip fracture; broader hip fracture	E: outcome-specific only; R: NI; F: limited broader/periprosthetic evidence	(29, 33, 34)
Physical frailty/sarcopenia	Strength, mobility, body composition	Functional recovery/rehabilitation	Direct THA plus mixed TJA	E: observational; R/F: NI. Definitions and thresholds vary	(10, 11, 18, 19, 22)
Dynamic/ML models	Longitudinal clinical variables	Trajectory updates	Single-centre hip replacement; mixed revision modelling	E: internal only; R: mixed-joint modelling; F: NI. No independent validation	(15, 35)

CFS, clinical frailty scale; CGA, comprehensive geriatric assessment; E, elective primary; F, fracture-related; FI, frailty index; HFRS, hospital frailty risk score; ICD, international classification of diseases; mFI, modified Frailty Index; NI, not identified; R, revision; RAI, risk analysis index; THA, total hip arthroplasty; TKA, total knee arthroplasty. Direct use or association is not equivalent to external validation. ‘Limited’ indicates incomplete independent pathway-specific calibration, threshold-stability, or net-benefit evidence; NI means that no pathway-specific external-validation study was found in the evidence reviewed. ML, machine learning.

## 3 Context-Specific interpretation

### 3.1 Elective primary THA

Elective primary THA offers the clearest opportunity to establish baseline mobility, cognition, nutrition, living circumstances, and patient goals before operative stress. Observational cohorts consistently associate greater frailty with complications, mortality, revision, and health-care use (5, 16, 36), while severe sarcopenia has been linked to slower recovery and non-home discharge (18). Preoperative time can support medication review, anaemia and nutritional assessment, feasible exercise, goal clarification, and discharge planning.

This pathway is also affected by health selection. Patients who reach elective THA have already passed referral, willingness, medical evaluation, and surgical-selection filters; the most vulnerable candidates may have been deferred, declined surgery, or never referred. Observed frailty prevalence and absolute mortality are influenced by this selection process. Linked routine-data analyses have identified a residual healthy-selection effect even after adjustment (36). Optimization should target plausible mechanisms without creating indefinite delay or turning a frailty score into an access barrier.

### 3.2 Revision THA

Revision THA combines patient vulnerability with indication-specific procedural complexity. Infection, instability, fracture, aseptic loosening, and implant failure involve different operative duration, blood loss, bone loss, restrictions, and recovery trajectories. Frailty measures have been associated with complications, non-home discharge, readmission, mortality, length of stay, and costs after revision arthroplasty (13, 31, 37–39). Much of this evidence combines revision THA and TKA; it should not be represented as a hip-specific effect when the subgroup is not separable. A 2026 systematic review of revision THA/TKA reached a similar overall conclusion but emphasized retrospective designs, mixed-joint cohorts, and the absence of a universally preferred tool (40).

Septic and aseptic revision may also modify the meaning of a given score. Infection adds inflammatory burden, antimicrobial treatment, repeated procedures, and prolonged functional disruption, whereas mechanical revision may produce a different pathway (31, 41). Models that include revision indication and procedural complexity may characterize operative risk more accurately than patient-level frailty alone. A high administrative or deficit score is most useful when it triggers domain assessment, blood-management planning, realistic rehabilitation goals, and enhanced surveillance.

### 3.3 Fracture-related arthroplasty

Arthroplasty after femoral-neck fracture occurs under time pressure. Pain, inflammation, immobility, dehydration, delirium, medication exposure, and nutritional deterioration may alter measurements intended to represent prefracture reserve. Informant history, prefracture mobility, residence, activities of daily living, cognition, and caregiver support are therefore essential for distinguishing baseline vulnerability from acute injury effects. A recent retrospective THA cohort found that several preoperative frailty scores were associated with two-year mortality, but the selected surgical population and wide confidence intervals do not justify a universal THA-vs.-hemiarthroplasty cutoff (42).

Direct fracture-arthroplasty evidence illustrates both the value and limits of abbreviated tools. In ACS-NSQIP data, mFI-5 predicted several morbidity and readmission outcomes after hemiarthroplasty and THA, but did not provide a uniformly strong mortality signal (4). Broader hip-fracture evidence supports multidimensional assessment, although not every included study involved a separable arthroplasty population (6). The acute pathway narrows the optimization window but does not eliminate action: delirium susceptibility, malnutrition, anaemia, dependence, and inadequate support can be identified rapidly while surgery proceeds without avoidable delay. Frailty contributes to these decisions alongside fracture pattern, prefracture function, life expectancy, patient goals, and surgical considerations.

The timing and meaning of frailty assessment differ across elective primary, revision, and emergency pathways (1, 2, 4, 5, 27, 37, 43). Figure 2 aligns the assessment points, intervention windows, and recovery milestones of the three trajectories on a common timeline. Elective primary THA emphasizes characterization and optimization; revision THA requires vulnerability to be combined with indication and complexity; and fracture arthroplasty requires rapid reconstruction of prefracture reserve. Pooling these trajectories requires explicit consideration of tool content, population selection, intervention window, and target outcome.

**Figure 2:**
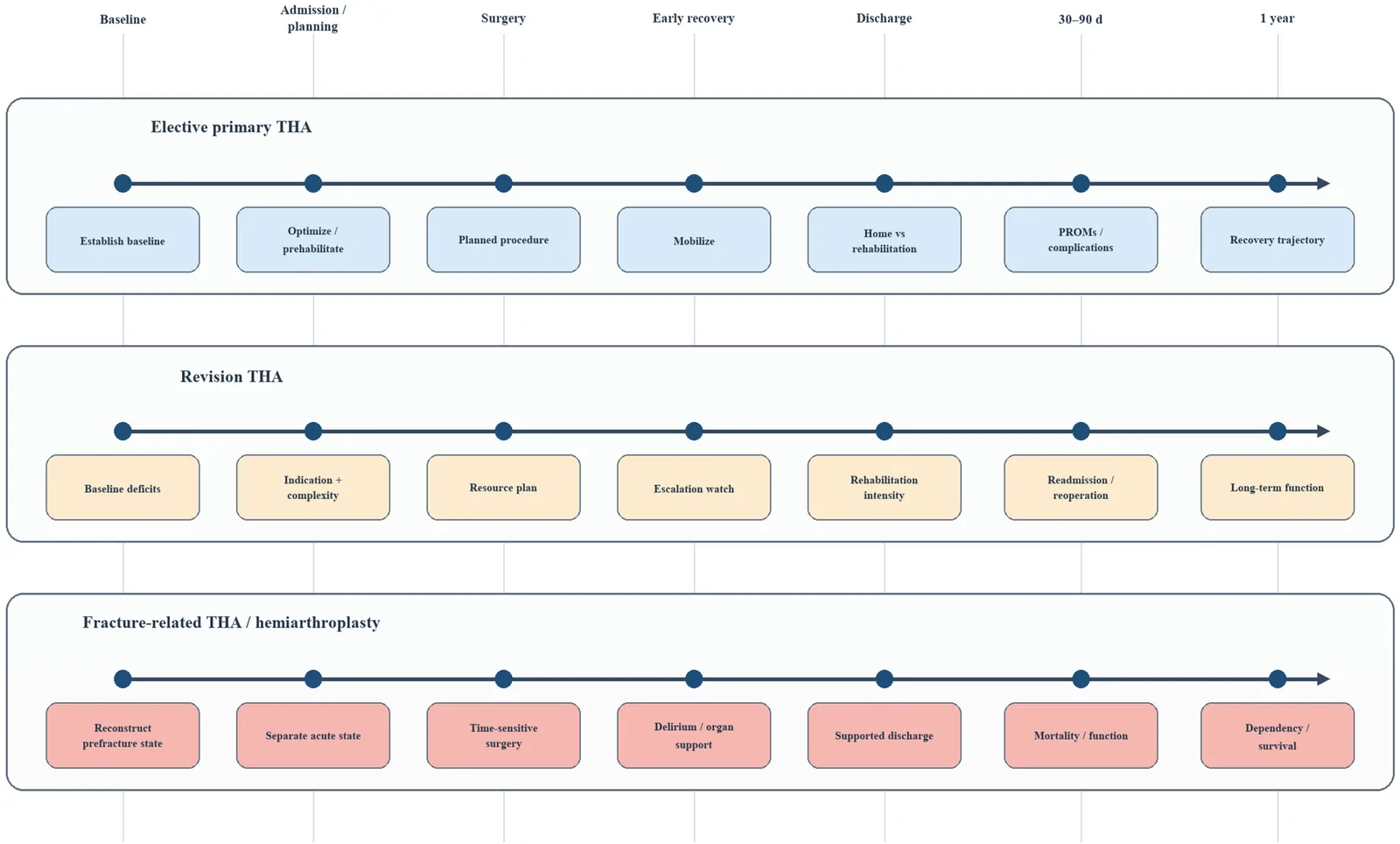
Time-sensitive frailty assessment across arthroplasty contexts. The timeline contrasts assessment, feasible intervention windows, early recovery, discharge, and longer-term outcomes in **(A)** elective primary THA, **(B)** revision THA, and **(C)** emergency THA or hemiarthroplasty. Context modifies baseline-risk reconstruction, measurement meaning, and feasible intervention; the proposed time points are not validated protocol requirements. Original figure created for this review from a multi-study synthesis (1, 2, 4, 5, 27, 37, 43). PROMs, patient-reported outcome measures; THA, total hip arthroplasty.

Meaningful reassessment depends on pathway and purpose. Elective patients can be assessed at surgical decision or preoperative clinic and again if health changes before surgery. Revision patients require reassessment when indication, infection status, or operative plan changes. In fracture pathways, admission scores should be paired with reconstructed prefracture function because acute inflammation and immobility can transiently worsen performance. Early postoperative review detects delirium, organ support, intake, and mobility; discharge review records destination and support; 30–90-day and longer-term follow-up evaluates PROMs, days at home, independence, and goal attainment. These time points are proposed for testing rather than validated requirements.

## 4 Outcomes that matter

### 4.1 Complications, return events, and survival

Across hip-arthroplasty cohorts, frailty is consistently associated with adverse postoperative events. A systematic review and meta-analysis of seven retrospective cohorts reported associations with total complications, reoperation, readmission, and 30-day mortality, while identifying substantial heterogeneity (14). Across studies, the direction of association is more consistent than its magnitude. Differences in frailty definition, population selection, primary vs. revision surgery, follow-up, and outcome coding limit direct numerical comparison.

Primary THA studies provide complementary evidence. Increasing mFI-5 severity has been associated with stepwise increases in short- and longer-term complications (16), and frailty combined with hypoalbuminaemia identified a group with a broader 30-day morbidity burden (5). In fracture arthroplasty, mFI-5 associations differed across procedures and individual outcomes (4). Reoperation and readmission capture different events. Reoperation more often reflects surgical or implant problems, whereas readmission may follow medical decompensation, inadequate support, rehabilitation barriers, or complications detected after discharge.

Mortality also depends on the time window and selection process. Frailty has been associated with short-term mortality after primary and revision arthroplasty (31, 35, 36) and with one-year mortality in population-based TJA data (2). A systematic review and meta-analysis of lower-limb arthroplasty also found higher mortality among patients classified as frail, although instrument and procedure heterogeneity limit direct translation to a single THA threshold (44). Longer follow-up increasingly reflects chronic disease and general health trajectories as well as surgery. Interpretation is improved by reporting calibrated absolute risk and acknowledging uncertainty around large estimates with wide confidence intervals. Figure 3 links distinct vulnerability domains with candidate perioperative processes and safety, functional, return-event, and resource outcomes (2, 5, 6, 14, 18, 20, 22, 23, 25). The arrows denote evidence-supported or plausible relationships and do not establish causality.

**Figure 3:**
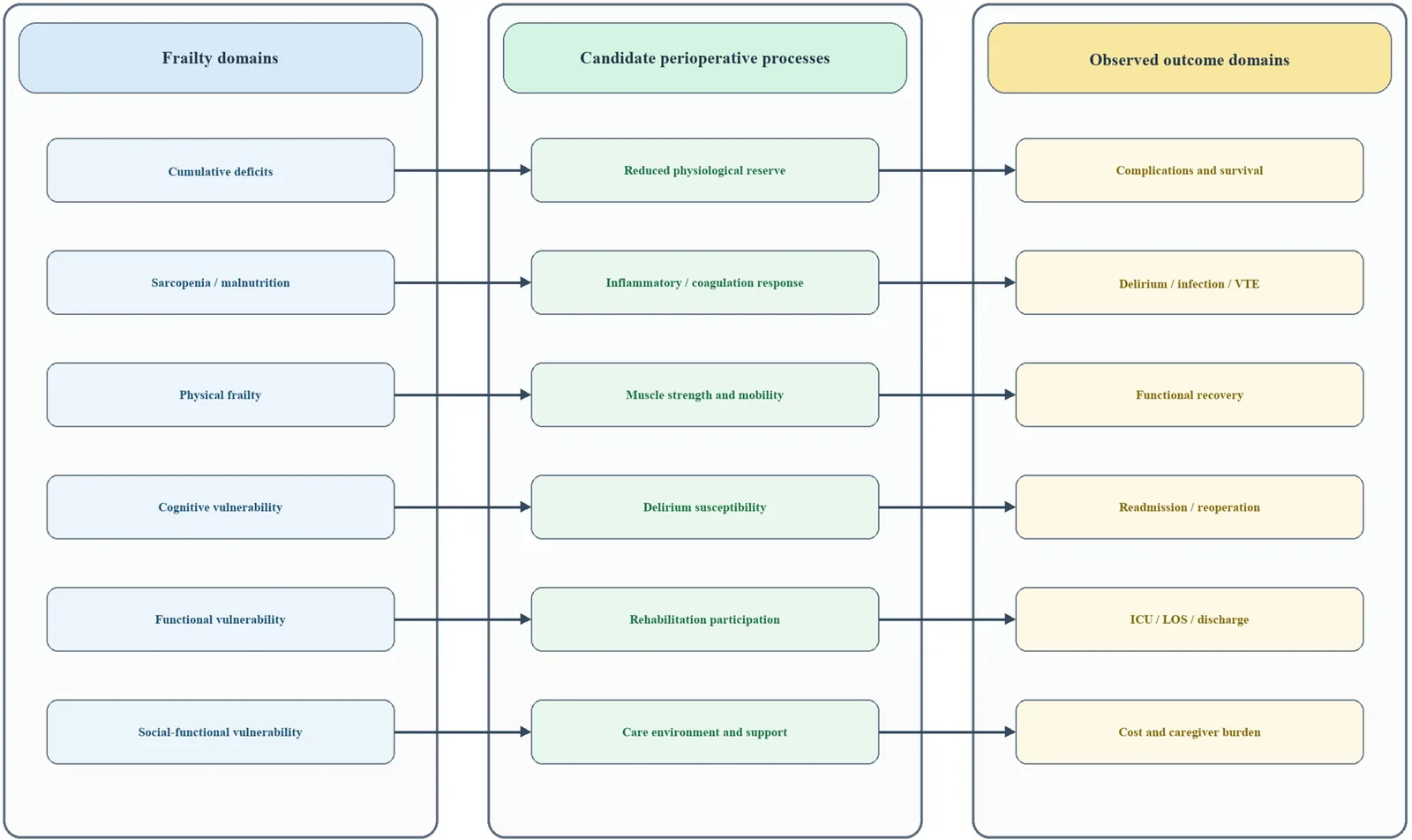
Vulnerability-domain-to-outcome pathways in hip arthroplasty. **(A)** Frailty-related domains are linked to **(B)** candidate perioperative processes and **(C)** observed safety, functional, return-event, and resource outcomes. Similar total scores may therefore represent different recovery trajectories. Arrows indicate supported or plausible associations but do not establish mediation, causation, or intervention effectiveness. Original figure created for this review from a multi-study synthesis (2, 5, 6, 14, 18, 20, 22, 23, 25). ICU, intensive care unit; LOS, length of stay; VTE, venous thromboembolism.

Delirium deserves separate attention because it is both an event and a marker of impaired cognitive reserve. Its interpretation should incorporate baseline cognition, education, sensory impairment, psychoactive medication, pain, sleep disruption, and acute illness rather than attribute the event solely to a frailty total score (23, 25). Reported associations with infection, pneumonia, venous thromboembolism, renal injury, and other complications also require outcome-specific definitions; composite outcomes can be driven by frequent minor events and administrative coding may not capture severity (16, 45, 46). Table 2 distinguishes direct, mixed-joint, context-specific, and suggestive evidence across vulnerability and outcome domains.

**Table 2:** Evidence-context matrix linking vulnerability domains with outcomes.

Clinical context	Tool/domain	Outcome	Evidence context	Main finding and limitation	References
Hip arthroplasty	Multiple frailty tools	Complications, reoperation, readmission, mortality	Direct but heterogeneous hip-arthroplasty cohorts	Associations broadly consistent; constructs and outcomes heterogeneous	(14)
Elective primary THA	Frailty + hypoalbuminaemia	30-day complications/mortality	Direct THA	Combined vulnerability associated with higher risk; observational residual confounding	(5)
Fracture THA/hemiarthroplasty	mFI-5	Morbidity, readmission, mortality	Direct fracture arthroplasty	Morbidity signal more consistent than mortality; observational	(4)
Hip-fracture surgery	Frailty + CALLY/GINI	In-hospital to 1-year mortality	Indirect hip-fracture; arthroplasty subgroup unavailable	AUC gain 0.666 to 0.678; severe collinearity and no external validation	(48)
Older hip arthroplasty	Frailty + cognitive vulnerability	Early delirium	Direct hip arthroplasty	Association only; limited observational evidence	(23)
Elective THA/TKA	mFI	Death/ICU admission	Mixed THA/TKA	One threshold missed many events; no separable hip estimate	(1)
Population TJA	Frailty	ICU, LOS, mortality, resources	Mixed TJA	Higher adjusted use/mortality; transferability to THA is incomplete	(2)
THA recovery	Sarcopenia/physical frailty	Function/recovery	Direct THA plus mixed-joint intervention	Worse recovery; intervention effects vary by endpoint	(18, 22, 43)
Revision arthroplasty	Frailty indices	Complications, LOS, readmission, mortality	Mostly mixed revision THA/TKA	Higher risk; tool performance differs; hip-specific estimates often unavailable	(31, 37, 40, 41)

CFS, clinical frailty scale; mFI, modified frailty index; THA, total hip arthroplasty; TJA, total joint arthroplasty; TKA, total knee arthroplasty. Direct THA, fracture-arthroplasty, mixed THA/TKA, and indirect hip-fracture evidence are labelled explicitly. Association does not establish that modifying a domain or applying a score-guided intervention improves outcomes. AUC, area under the receiver operating characteristic curve; CALLY, C-reactive protein-albumin-lymphocyte index; GINI, Global Immuno-Nutrition Inflammation index; ICU, intensive care unit; LOS, length of stay.

### 4.2 Functional recovery and rehabilitation

Functional recovery is a central outcome for many older adults. Early recovery depends on strength, gait, cognition, pain, haemoglobin, motivation, and support. Prospective mixed THA/TKA evidence suggests that preoperative frailty may be associated with lower early Functional Independence Measure scores, although the small sample limits inference (43). Direct THA evidence more clearly links severe sarcopenia with delayed recovery, non-home discharge, and poorer patient-reported outcomes (18). Disposition should be interpreted relative to baseline residence, goals, caregiver capacity, and local rehabilitation supply.

Studies use several non-equivalent definitions of recovery, including return to baseline function, clinically important improvement, independent living, and attainment of an individual goal. Hip scores and patient-reported outcomes may improve while walking independence remains limited, and body-composition gains may occur without better activities of daily living (22). Studies should therefore report preoperative or prefracture status and repeated measures rather than a single postoperative cross-section.

Longitudinal modelling illustrates the potential of dynamic risk estimation. A single-centre study used repeated clinical variables to estimate postoperative frailty risk and reported time-dependent discrimination, calibration, decision curves, and feature contributions (15). The study demonstrates feasibility but not clinical effectiveness; internal validation, data augmentation, and a single-centre sample require independent external validation. Machine-learning models also require assessment of overfitting, calibration drift, transportability, subgroup fairness, missing-data sensitivity, explainability, and decision-curve net benefit before implementation. Discrimination alone does not establish clinical utility. Likewise, prehabilitation utilization reflects both need and access and cannot be interpreted as evidence of efficacy (26).

### 4.3 Intensive care and resource use

Population-based mixed TJA data associate frailty with intensive care admission, mortality, longer hospitalization, and greater costs (2). These outcomes are partly process-dependent. Planned postoperative monitoring, unplanned deterioration, organ support, and intensive-care length of stay have different clinical meanings. The Danish registry in which most patients with death or intensive-care admission did not cross the mFI threshold illustrates why an abbreviated comorbidity score should not be the sole criterion for escalation (1). Acute physiology, cognition, mobility, procedure type, and local practice remain essential.

Length of stay and discharge destination similarly reflect interactions between patient need and health-system capacity. Revision complexity, complications, baseline dependence, rehabilitation availability, transport, equipment, and placement all contribute (2, 37, 39). Cost evidence should specify perspective, time horizon, inflation adjustment, and inclusion of post-acute care. High expenditure may indicate preventable complications, but it may also represent appropriate investment in recovery. Used prospectively, risk information may help coordinate monitored beds, geriatric input, rehabilitation placement, caregiver preparation, and follow-up.

## 5 From prediction to action

### 5.1 A layered assessment pathway

A practical pathway begins with a low-burden screen of comorbidity and dependence, usual mobility, cognition or delirium risk, nutrition, living situation, and caregiver support. Screening may be completed by the admitting nurse or clinician at preoperative clinic or emergency admission. A positive screen, a proposed local trigger, or discordance between a low score and obvious vulnerability should prompt targeted assessment by the responsible clinician and, where available, geriatric, nutrition, pharmacy, or rehabilitation staff. The purpose is to define clinically relevant vulnerability domains and decisions, not to assign a treatment solely from a score.

The second layer combines vulnerability domains with procedure context and target outcome. Elective primary THA permits preoperative assessment and optimization; revision THA adds indication and procedural complexity; fracture arthroplasty requires rapid reconstruction of prefracture status without avoidable surgical delay. Figure 4 specifies illustrative assessors, timing, escalation triggers, responsibilities, and reassessment points (1, 6, 15, 22, 26, 41, 47). Postoperative, discharge, 30–90-day, and longer-term updates use mobility, cognition, nutrition, complications, PROMs, days at home, caregiver capacity, and goal attainment. These elements remain a conceptual workflow requiring prospective validation.

**Figure 4:**
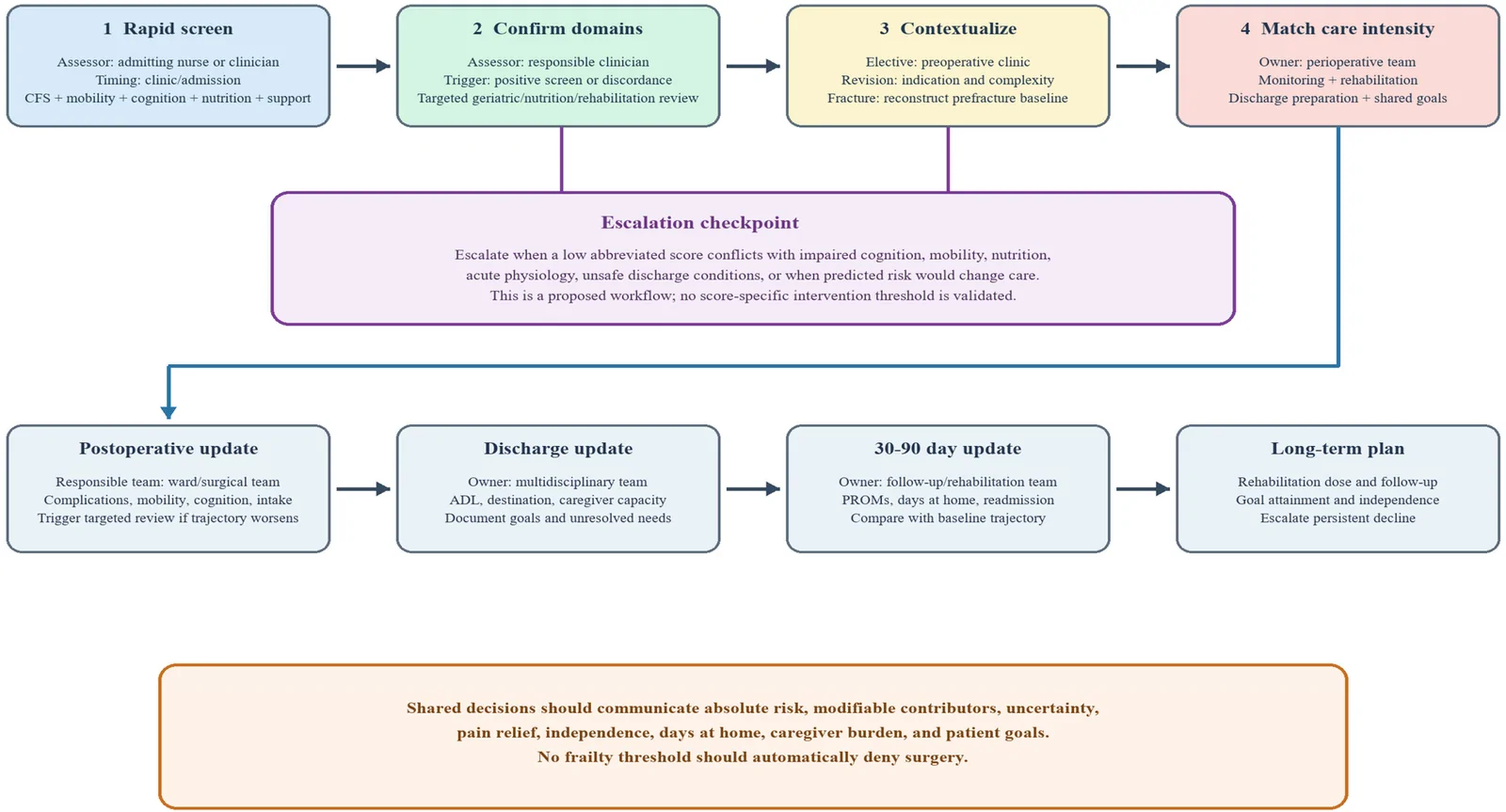
Proposed operational pathway from frailty screening to dynamic recovery planning. Screening may be performed by an admitting nurse or clinician in preoperative clinic or at emergency admission; a positive screen or discordance between the score and cognition, mobility, nutrition, acute physiology, or discharge safety triggers targeted review by the responsible clinician and available geriatric, nutrition, pharmacy, and rehabilitation staff. The multidisciplinary team documents context, care responsibilities, patient goals, and reassessment after surgery, at discharge, at 30–90 days, and during longer-term recovery. This is an illustrative conceptual workflow, not a validated clinical algorithm or a set of mandatory score thresholds. Original figure created for this review from a multi-study synthesis (1, 6, 15, 22, 26, 41, 47). ADL, activities of daily living; CFS, clinical frailty scale; PROMs, patient-reported outcome measures.

A tiered strategy may be more feasible in high-volume or resource-constrained settings. A minimum tier uses a brief bedside score, usual mobility, cognition, oral intake or weight loss, and living support, relying on routine observations rather than specialized testing. A targeted tier adds domain-specific tests or routine laboratory markers only when the result could alter monitoring, nutrition, rehabilitation, discharge, or shared decisions. A full multidomain or geriatric assessment is reserved for complex or discordant cases where trained staff are available. Where specialist geriatric, laboratory, or rehabilitation capacity is limited, the responsible surgical or perioperative team should document the unmet need, apply low-burden measures, and use explicit referral or follow-up criteria rather than omit assessment entirely.

### 5.2 Domain-informed care and communication

Cumulative-deficit vulnerability may prompt medication review, chronic-disease and anaemia management, and closer surveillance (49). Physical or sarcopenic vulnerability may support nutrition assessment, progressive resistance work, early mobilization, and dose-defined rehabilitation (18, 22). Cognitive vulnerability should activate delirium-prevention measures, sensory support, sleep and pain management, and caregiver involvement (23). Social-functional vulnerability requires early discharge planning, equipment, transport, rehabilitation access, and transitional follow-up. These are evidence-supported general practices or biologically plausible responses, not validated score-specific treatments. Table 3 separates associative, mechanistic, interventional, and hypothesis-generating support and identifies illustrative responsibilities and escalation points.

**Table 3:** Domain-informed perioperative action matrix with evidence classification and operational responsibilities.

Domain/signal	Evidence type	Preoperative/admission action	Early recovery/follow-up	Assessor, timing, escalation	References
Cumulative deficits: high FI/mFI/HFRS	Associative; hypothesis-generating pathway	Medication/chronic disease review; establish baseline	Enhanced surveillance; medication reconciliation	Nurse/clinician at clinic/admission; escalate discordance or care-changing risk	(4, 13, 47)
Physical/sarcopenic: weakness or low mobility	Associative; domain-targeted intervention evidence only; no validated frailty-guided pathway	Nutrition assessment; feasible prehabilitation	Dose-defined mobilization/resistance; trajectory review	Rehabilitation/clinical team; assess before surgery and after mobility change	(18, 19, 22)
Nutritional/inflammatory: weight loss, anaemia, low intake, abnormal routine markers	Associative/mechanistic; adjunct prediction	Identify reversible causes without unsafe delay	Monitor intake, inflammation context, and recovery	Clinician/dietitian where available; escalate severe or discordant findings	(5, 48)
Cognitive: impairment/delirium risk	Associative + evidence-supported general practice	Document baseline and caregiver history	Delirium prevention; supported communication	Nurse/clinician at admission and daily if acute change	(23, 25)
Social-functional: dependence/limited support	Associative; service intervention plausible	Early discharge-needs and goals assessment	Caregiver engagement; home/facility rehabilitation	Multidisciplinary team from admission to discharge; escalate unsafe destination	(6, 12)
Low score with domain-specific risk	Hypothesis-generating	Targeted multidomain review	Do not down-triage solely by abbreviated score	Responsible clinician; trigger is score-clinical discordance	(1, 6)

Evidence type describes support for each row, not proof that a score-specific intervention improves outcomes. Associative evidence links a domain with an outcome; mechanistic rationale is biologically or clinically plausible; interventional evidence tests an action but may not be frailty-stratified. Physical/sarcopenic intervention evidence targets related nutrition or rehabilitation domains and does not validate a frailty-guided care pathway. Hypothesis-generating actions require prospective evaluation. CFS, clinical frailty scale; FI, frailty index; HFRS, Hospital frailty risk score; mFI, modified frailty index.

Risk communication should describe dominant sources of vulnerability, calibrated absolute risk when available, potentially modifiable contributors, and uncertainty. Patient-centred outcomes should include pain relief, independence, return to baseline function, days at home, caregiver burden, PROMs, and individualized goal attainment. These outcomes may be valued differently from survival or complication avoidance. Higher vulnerability may justify more support and monitoring, not less access. If surgery is unlikely to achieve a patient's goals, that conclusion should follow multidisciplinary assessment and shared decision-making rather than a rigid frailty threshold.

## 6 An integrated framework and research priorities

Taken together, the evidence is best read as four linked layers rather than as a new scoring system. The domain layer describes accumulated deficits, physical reserve, nutrition and sarcopenia, cognition and emotion, and social-functional capacity. The context layer places these domains within elective primary, revision, fracture THA, or hemiarthroplasty pathways and records timing and stress intensity. The outcome layer distinguishes complications and survival from recovery trajectories, return events, patient goals, and resource use, while the action layer connects findings with optimization, monitoring, rehabilitation, and transitional care. This framework is an analytic synthesis, not a validated score or algorithm.

Three near-term studies deserve priority. First, a geographically diverse external-validation study should compare simple tools across elective primary, revision, and fracture-related pathways, reporting calibration-in-the-large, calibration slope, subgroup performance, threshold consequences, and decision-curve net benefit. Second, a pragmatic cluster or stepped-wedge trial should compare a tiered domain-informed pathway with usual care using days at home, goal attainment, independence, and major complications as co-primary or hierarchically ordered outcomes. Third, a multicentre trajectory cohort should repeat frailty-domain assessment from baseline or reconstructed prefracture status through discharge and 30–90 days to identify clinically meaningful recovery states. These priorities are proposals from this synthesis, not a consensus ranking.

A minimum frailty dataset could improve comparability without stripping away context. Reports should include instrument name and version, component domains, cut-points, assessment timing, missing-data rules, an abbreviated deficit measure, mobility or physical performance, cognition and delirium risk, nutrition and inflammatory context, baseline or prefracture function, residence and support, procedure type, and revision indication. Outcomes should distinguish major complications, delirium, time-specific survival, return to baseline function, independent living, PROMs, goal attainment, days at home, caregiver burden, readmission or reoperation, planned and unplanned intensive care, rehabilitation dose, and costs.

Sample size should be planned for each design rather than inferred from this narrative review. Prediction studies should use anticipated event rates and precision targets for calibration slope and external-validation performance, with allowance for missing data and planned subgroup analyses. Cluster or stepped-wedge trials should use a prespecified clinically important difference, intracluster correlation, cluster-size variation, attrition, and multiplicity assumptions. No universal sample-size value is proposed because the necessary event-rate and effect-size inputs are not established. Across studies, age, sex, race or ethnicity where legally and ethically collected, socioeconomic context, geography, healthcare system, access to rehabilitation, and missingness should be reported and examined without assuming transportability. Table 4 summarizes these actionable priorities and their methodological requirements.

**Table 4:** Actionable near-term research priorities and methodological requirements.

Priority/question	Population/comparator	Design	Primary outcomes	Sample-size basis	Minimum dataset/decision target	Rank
Context-specific tool validation	Elective primary, revision, and fracture pathways; simple baseline vs. candidate tool	Geographically diverse external validation	Calibration slope/in-the-large, net benefit, major outcomes	Event rate + precision targets; allow missingness/subgroups	Instrument version, domains, timing, context, equity strata; model selection	1
Does tiered domain-informed care improve recovery?	Eligible older arthroplasty patients; pathway vs. usual care	Pragmatic cluster or stepped-wedge trial	Days at home, goal attainment, independence, major complications	Clinically important difference + cluster design effect + attrition	Fidelity, cointerventions, PROMs, caregiver burden; care-pathway decision	2
Which trajectories matter?	Elective/revision/fracture cohorts; repeated vs. baseline-only assessment	Prospective multicentre trajectory cohort	Recovery states at discharge and 30–90 days	State prevalence/transition precision + attrition	Baseline/prefracture function, repeated domains, rehabilitation dose; reassessment timing	3
Resource implementation	High-volume/resource-constrained sites; minimum vs. expanded tier	Hybrid implementation-effectiveness study	Feasibility, equity, unmet need, patient outcomes	Site-level precision and implementation contrast	Staffing, lab/rehabilitation access, referral completion; scalable workflow	-
ML clinical utility	Independent institutions/time periods; simple model comparator	Temporal/geographic validation + impact study	Calibration drift, fairness, decision-curve net benefit	External event count and subgroup precision	Missingness, explainability, thresholds; deployment decision	-

ML, machine learning; PROMs, patient-reported outcome measures. The proposed priorities and planning principles are research recommendations, not validated sample-size requirements or clinical implementation standards.

## 7 Limitations

The evidence base is dominated by retrospective cohorts, administrative databases, and studies that combine THA and TKA. Frailty definitions, thresholds, coding, procedure categories, outcome windows, and health-system context vary substantially. Residual confounding and selection bias are unavoidable, especially in elective surgery. Broader hip-fracture studies may include internal fixation or non-separable procedures and were treated as indirect evidence unless arthroplasty was explicit. The Karahan study also included hip-fracture surgery rather than a separable arthroplasty cohort and therefore informs adjunct mortality prediction, not direct arthroplasty treatment effects (48).

Functional, social, caregiver, and long-term recovery outcomes are reported less consistently than complications and mortality. Searches were restricted to PubMed and Web of Science, and non-English or incompletely indexed evidence may have been under-represented. Grey literature, registries, and conference abstracts were excluded as primary evidence because methods, peer review, and complete outcome data were often unavailable; this may preferentially omit negative, preliminary, or unpublished findings and may inflate apparent associations. This framework-oriented narrative synthesis did not use a uniform risk-of-bias instrument or meta-analysis, so comparative certainty and publication-bias magnitude cannot be quantified. Proposed domain-informed actions remain partly inferential because direct trials are scarce.

Figures 1–4 are original conceptual syntheses created for this review and do not reproduce or adapt published image blocks. Their arrows, pathways, assessors, triggers, and action mappings summarize heterogeneous evidence and illustrative workflow choices; neither the causal structure nor any score-specific treatment pathway has been validated.

## 8 Conclusions

Frailty helps explain why recovery and complications vary after hip arthroplasty, but no single score captures that variation. Cumulative deficits, physical reserve, nutrition and inflammatory context, cognition, and social-functional capacity interact with elective primary, revision, or fracture-related surgery. The practical value of assessment lies in making the vulnerability domain, context, target outcome, evidence type, and possible response explicit.

Research should prioritize context-specific external validation, longitudinal recovery measurement, and pragmatic evaluation of tiered domain-informed care. Until such evidence is available, frailty assessment should support proportional monitoring, rehabilitation, discharge planning, and shared decisions; it should not be used as a mechanical barrier to surgery or as proof that a proposed intervention will improve outcomes.

## References

[1] Laursen CC Meyhoff CS Petersen TS Jimenez-Solem E Sorensen AMS Lunn TH. Fatal outcome and intensive care unit admission after total hip and knee arthroplasty: an analytic of preoperative frailty and comorbidities. Acta Anaesthesiol Scand. (2021) 65:1390–6. 10.1111/aas.13950 34252199

[2] McIsaac DI Beaule PE Bryson GL Van Walraven C. The impact of frailty on outcomes and healthcare resource usage after total joint arthroplasty: a population-based cohort study. Bone Jt J. (2016) 98-B:799–805. 10.1302/0301-620X.98B6.37124 27235523

[3] Hung M Jensen A Strickler I Vu S Hon ES Arapovic A. Frailty and sarcopenia as predictors of functional recovery after total hip and knee arthroplasty: a narrative review. J Clin Med. (2026) 15:3578. 10.3390/jcm15103578 42194536 PMC13207190

[4] Kim AG Grits D Zhong J Chiu AM Reading L Zuke W. 5-Factor Modified frailty Index as a predictor of outcomes after hemiarthroplasty or total hip arthroplasty for femoral neck fracture. J Am Acad Orthop Surg. (2024) 32:e634–41. 10.5435/JAAOS-D-23-00936 38569220

[5] Wilson J Schwartz A Farley K Bradbury T Guild G. Combined malnutrition and frailty significantly increases complications and mortality in patients undergoing elective total hip arthroplasty. J Arthroplasty. (2020) 35:2488–94. 10.1016/j.arth.2020.04.028 32444236

[6] Li Y Sun X. Association between multidimensional frailty and postoperative outcomes in older patients with hip fracture: a scoping review. J Am Med Dir Assoc. (2025) 26:105769. 10.1016/j.jamda.2025.105769 40651500

[7] Searle SD Mitnitski A Gahbauer EA Gill TM Rockwood K. A standard procedure for creating a frailty index. BMC Geriatr. (2008) 8:24. 10.1186/1471-2318-8-24 18826625 PMC2573877

[8] Gilbert T Neuburger J Kraindler J Keeble E Smith P Ariti C. Development and validation of a hospital frailty risk score focusing on older people in acute care settings using electronic hospital records: an observational study. Lancet. (2018) 391:1775–82. 10.1016/S0140-6736(18)30668-8 29706364 PMC5946808

[9] Rockwood K Song X MacKnight C Bergman H Hogan DB McDowell I. A global clinical measure of fitness and frailty in elderly people. CMAJ. (2005) 173:489–95. 10.1503/cmaj.050051 16129869 PMC1188185

[10] Fried LP Tangen CM Walston J Newman AB Hirsch C Gottdiener J. Frailty in older adults: evidence for a phenotype. J Gerontol A Biol Sci Med Sci. (2001) 56:M146–56. 10.1093/gerona/56.3.M146 11253156

[11] Cruz-Jentoft AJ Bahat G Bauer J Boirie Y Bruyère O Cederholm T. Sarcopenia: revised European consensus on definition and diagnosis. Age Ageing. (2019) 48:16–31. 10.1093/ageing/afy169 30312372 PMC6322506

[12] Krishnan M Beck S Havelock W Eeles E Hubbard R Johansen A. Predicting outcome after hip fracture: using a frailty index to integrate comprehensive geriatric assessment results. Age Ageing. (2014) 43:122–6. 10.1093/ageing/aft084 23832264

[13] Meyer M Schwarz T Renkawitz T Maderbacher G Grifka J Weber M. Hospital frailty risk score predicts adverse events in revision total hip and knee arthroplasty. Int Orthop. (2021) 45:2765–72. 10.1007/s00264-021-05038-w 33860337 PMC8560670

[14] Wen H Liu T Li J. Association between frailty and clinical post-operative outcomes in patients following hip arthroplasty: a systematic review and meta-analysis. Int Orthop. (2023) 47:667–75. 10.1007/s00264-022-05657-x 36542141

[15] Lv X Li H Li Y Zhuo R Yue Y Wang Y. Dynamic frailty risk prediction in elderly hip replacement: a deep learning approach to personalized rehabilitation. BMC Med Inform Decis Mak. (2025) 25:292. 10.1186/s12911-025-03143-z 40770716 PMC12330151

[16] Tummala S Mittal MM Chavda H Sontam TR Sambandam SN Wukich DK. The influence of frailty on total hip arthroplasty outcomes: a propensity-matched study of 90,660 patients using the modified frailty Index. J Clin Med. (2026) 15:2802. 10.3390/jcm15072802 41977104 PMC13073173

[17] Ali U Malik SAR Iqbal B Bhatti A Khan SB Noordin S. The modified 5-item frailty index in total hip arthroplasty patients: a retrospective cohort from a low-middle income country. J Orthop Surg. (2025) 20:299. 10.1186/s13018-025-05505-9 PMC11924793 40108605

[18] Tanaka S Kayamoto A Terai C Nojiri S Fugane Y Mori T. Preoperative sarcopenia severity and clinical outcomes after total hip arthroplasty. Nutrients. (2024) 16:2085. 10.3390/nu16132085 38999833 PMC11243643

[19] Su Y Peng L Dong D Ma Z Gu X. Impact of sarcopenia in elderly patients undergoing elective total hip arthroplasty on postoperative outcomes: a propensity score-matched study. BMC Anesthesiol. (2024) 24:158. 10.1186/s12871-024-02538-1 38658828 PMC11040774

[20] Okamoto Y Wakama H Saika T Tani K Otsuki S. Osteosarcopenia as a predictor of postoperative adverse outcomes in older adults undergoing total hip arthroplasty. J Bone Miner Metab. (2026) 44:114–23. 10.1007/s00774-025-01671-2 41339538

[21] Sumbal R Abbas M Sheikh SM Sumbal A. Prevalence and clinical impact of sarcopenia in patients undergoing total joint arthroplasty: a systematic review and a meta-analysis. J Arthroplasty. (2024) 39:3128–3135.e3. 10.1016/j.arth.2024.06.021 38901711

[22] Han Z Ji N Ma J Dong Q Ma X. Effect of resistance training combined with Beta-hydroxy-Beta-methylbutyric acid supplements in elderly patients with sarcopenia after hip replacement. Orthop Surg. (2022) 14:704–13. 10.1111/os.13208 35195339 PMC9002065

[23] Ma Z Wang J He T Zhu S Sheng C Ge Y. Correlation between preoperative frailty and postoperative delirium in elderly patients undergoing hip arthroplasty. Medicine (Baltimore). (2023) 102:e34785. 10.1097/MD.0000000000034785 37653780 PMC10470691

[24] Evered LA Vitug S Scott DA Silbert B. Preoperative frailty predicts postoperative neurocognitive disorders after total hip joint replacement surgery. Anesth Analg. (2020) 131:1582–8. 10.1213/ANE.0000000000004893 33079882

[25] Jatti RS Manvi N Agarwal S. Cognitive impairment and postoperative outcomes in patients undergoing primary total hip arthroplasty and primary total knee arthroplasty: a systematic review and meta-analysis. Ann Afr Med. (2026). 10.4103/aam.aam_465_26 42411372 PMC13645293

[26] Stern BZ Sabo GC Balachandran U Agranoff R Hayden BL Moucha CS. Frailty is strongest need factor among predictors of prehabilitation utilization for total hip or knee arthroplasty in fee-for-service medicare beneficiaries. Phys Ther. (2025) 105:pzae183. 10.1093/ptj/pzae183 39714224

[27] Jo S Lee SH Yoon SJ. Clinical outcomes of total hip arthroplasty for displaced femoral neck fractures in patients 80 years of age and older selected by clinical frailty score. Hip Pelvis. (2020) 32:148–55. 10.5371/hp.2020.32.3.148 32953707 PMC7476787

[28] Wall BJ Wittauer M Dillon K Seymour H Yates PJ Jones CW. Clinical frailty scale predicts outcomes following total joint arthroplasty. Arthroplasty Lond Engl. (2025) 7:13. 10.1186/s42836-024-00294-8 PMC11874104 40025603

[29] Sasaki J Koltenyuk V Albert S Salehi H Sambare TD Andrawis J. The risk analysis index predicts 30-day periprosthetic fracture following primary total hip arthroplasty. J Orthop. (2025) 69:112–6. 10.1016/j.jor.2025.03.018 40212940 PMC11979430

[30] Traven SA Reeves RA Sekar MG Slone HS Walton ZJ. New 5-factor modified frailty Index predicts morbidity and mortality in primary hip and knee arthroplasty. J Arthroplasty. (2019) 34:140–4. 10.1016/j.arth.2018.09.040 30337252

[31] Zamanzadeh RS Seilern Und Aspang JRM Schwartz AM Martin JR Premkumar A Wilson JM. Age-Adjusted modified frailty Index predicts 30-day complications and mortality in aseptic revision total hip and knee arthroplasty. J Arthroplasty. (2024) 39:198–205. 10.1016/j.arth.2023.06.032 37380143

[32] Aspang J Zamanzadeh R Schwartz A Premkumar A Martin J Wilson J. The age-adjusted modified frailty Index: an improved risk stratification tool for patients undergoing primary total hip arthroplasty. J Arthroplasty. (2022) 37:1098–104. 10.1016/j.arth.2022.02.055 35189289

[33] Jowkar N Ruffner M Koltenyuk V Sokolik ID Horsley H Rohl M. Risk stratification in periprosthetic hip fracture surgery: the risk analysis Index is superior to the five-item modified frailty Index for predicting 30-day complications. J Arthroplasty. (2026) S0883–5403:00664–9. 10.1016/j.arth.2026.06.043 42341960

[34] Gupta NK Chmait HR Gill V Turnow M Manes T Taylor BC. Risk analysis Index for estimation of 30-day postoperative mortality in hip fractures. JAMA Netw Open. (2025) 8:e2512689. 10.1001/jamanetworkopen.2025.12689 40440016 PMC12123473

[35] Pean CA Buddhiraju A Shimizu MR Chen TLW Esposito JG Kwon YM. Prediction of 30-day mortality following revision total hip and knee arthroplasty: machine learning algorithms outperform CARDE-B, 5-item, and 6-item modified frailty Index risk scores. J Arthroplasty. (2024) 39:2824–30. 10.1016/j.arth.2024.05.056 38797444

[36] Cook M Lunt M Board T O’Neill T. The impact of frailty on short-term mortality following primary total hip and knee arthroplasty due to osteoarthritis. Age Ageing. (2022) 51:afac118. 10.1093/ageing/afac118 35679192 PMC9180921

[37] Traven SA Reeves RA Slone HS Walton ZJ. Frailty predicts medical complications, length of stay, readmission, and mortality in revision hip and knee arthroplasty. J Arthroplasty. (2019) 34:1412–6. 10.1016/j.arth.2019.02.060 30930155

[38] Johnson RL Abdel MP Frank RD Chamberlain AM Habermann EB Mantilla CB. Impact of frailty on outcomes after primary and revision total hip arthroplasty. J Arthroplasty. (2019) 34:56–64.e5. 10.1016/j.arth.2018.09.078 30340916

[39] Tram MK Tabbaa A Lakra A Anoushiravani AA Bernasek TL Lyons ST. Patient frailty is correlated with increased adverse events and costs after revision total hip arthroplasty. J Arthroplasty. (2024) 39:1151–1156.e4. 10.1016/j.arth.2023.12.027 38135165

[40] Braescu RD Pătrașcu JM Pătrașcu JM Cojocaru DG. The prognostic value of frailty assessment tools in predicting postoperative outcomes after revision total hip and knee arthroplasty: a systematic review. J Clin Med. (2026) 15:4489. 10.3390/jcm15124489 42355658 PMC13301404

[41] Grimmett H Sayyed A Koltenyuk V Areti A Chmait HR Gupta N. Comparative analysis of frailty indices on complication risk following septic revision total hip and knee arthroplasty. Geriatr Orthop Surg Rehabil. (2025) 16:21514593251392692. 10.1177/21514593251392692 41180942 PMC12572601

[42] Patton J Smith L Hoggett L Van Mierlo R McLauchlan G. Frailty assessment in total hip arthroplasty for femoral neck fracture. Hip Pelvis. (2026) 38:180–6. 10.5371/hp.2026.38.2.180 42226691 PMC13247327

[43] Yoshida I Sawaya Y Hiiragi Y Kikuchi S Urano T. Preoperative frailty in patients undergoing total knee or hip arthroplasty affects FIM scores at one week postoperatively. Nihon Ronen Igakkai Zasshi Jpn J Geriatr. (2025) 62:178–86. 10.3143/geriatrics.62.178 40582855

[44] Bai Y Zhang X-M Sun X Li J Cao J Wu X. The association between frailty and mortality among lower limb arthroplasty patients: a systematic review and meta-analysis. BMC Geriatr. (2022) 22:702. 10.1186/s12877-022-03369-w 35999509 PMC9400276

[45] Koltenyuk V Sasaki J Nowak K Fuller SI Kovacs AR Lucas TS. Frailty is associated with increased odds of 30-day periprosthetic joint infection following primary total hip arthroplasty. J Am Acad Orthop Surg. (2025). 10.5435/JAAOS-D-25-00840 41490324

[46] Lan C Mao Z Zhou Y Cai G Ren Z Hu Z. Impact of preoperative frailty on venous thromboembolism risk following total hip and knee arthroplasty: a meta-analysis. Int J Surg Lond Engl. (2025) 111:7155–67. 10.1097/JS9.0000000000002773 PMC12527706 40540311

[47] Ondeck NT Bohl DD Bovonratwet P Anandasivam NS Cui JJ McLynn RP. Predicting adverse outcomes after total hip arthroplasty: a comparison of demographics, the American society of anesthesiologists class, the modified charlson comorbidity Index, and the modified frailty Index. J Am Acad Orthop Surg. (2018) 26:735–43. 10.5435/JAAOS-D-17-00009 30130353

[48] Karahan M Ozdemir E Kina S. Integrated nutritional-inflammatory and frailty-based model for mortality risk stratification following hip fracture surgery: a multicentre cohort study. Aging Clin Exp Res. (2026) 38:95. 10.1007/s40520-026-03345-z 41731220 PMC12979323

[49] Gregersen M Borris LC Damsgaard EM. Postoperative blood transfusion strategy in frail, anemic elderly patients with hip fracture: the TRIFE randomized controlled trial. Acta Orthop. (2015) 86:363–72. 10.3109/17453674.2015.1006980 25586270 PMC4443456

