# Supplementary material for: Frailty across Hip arthroplasty pathways: vulnerability domains, outcomes, and implications for care—a narrative review
Source: Front Surg. 2026 Sep 22;13:1957876. doi: 10.3389/fsurg.2026.1957876 (PMC13640993; doi:10.3389/fsurg.2026.1957876)
Supplement: Supplementary file 1 [file Supplementaryfile1.docx]

Supplementary File 1. Search strategy and evidence-selection record

# Original database searches

PubMed and Web of Science Core Collection were searched from database inception through 27 July 2026. The original exports contained 235 PubMed and 233 Web of Science records (468 merged records; 362 unique records after cross-database deduplication). The complete original search strings and execution records are reported below.

## Original PubMed search

Database: PubMed

Search date: 27 July 2026

Coverage: Database inception through 27 July 2026; no publication-date restriction was applied in the search string.

Result and export count: 235 records. The PubMed ESearch identifier count and the PMID count in the raw MEDLINE/NBIB export were both 235.

Full search string (final executed B strategy):

("Arthroplasty, Replacement, Hip"[MeSH Terms]
 OR "total hip arthroplasty"[Title/Abstract]
 OR "total hip replacement"[Title/Abstract]
 OR "hip arthroplasty"[Title/Abstract])
AND
("Frailty"[MeSH Terms]
 OR "Frail Elderly"[MeSH Terms]
 OR frail*[Title/Abstract]
 OR "frailty index"[Title/Abstract]
 OR "modified frailty index"[Title/Abstract]
 OR mFI-5[Title/Abstract]
 OR mFI-11[Title/Abstract])
AND
("Aged"[MeSH Terms]
 OR "Aged, 80 and over"[MeSH Terms]
 OR "older adult"[Title/Abstract]
 OR "older adults"[Title/Abstract]
 OR elderly[Title/Abstract]
 OR geriatric*[Title/Abstract])
AND
(
 ("Intensive Care Units"[MeSH Terms]
 OR "Critical Care"[MeSH Terms]
 OR "intensive care unit"[Title/Abstract]
 OR "intensive care units"[Title/Abstract]
 OR ICU[Title/Abstract]
 OR "critical care"[Title/Abstract])
 OR
 ("Treatment Outcome"[MeSH Terms]
 OR "Prognosis"[MeSH Terms]
 OR "Postoperative Complications"[MeSH Terms]
 OR outcome*[Title/Abstract]
 OR prognos*[Title/Abstract]
 OR mortality[Title/Abstract]
 OR complication*[Title/Abstract]
 OR readmission*[Title/Abstract]
 OR "length of stay"[Title/Abstract])
)

## Original Web of Science Core Collection search

Database: Web of Science Core Collection

Search/export date: 27 July 2026

Coverage: Database inception through 27 July 2026; no publication-year restriction was applied in the search string.

Export count: 233 records in the preserved raw RIS file. The search-results-page hit count was not separately recorded; therefore, 233 is reported as the verified export count rather than as an independently verified page hit count.

Full search string (final recorded B strategy used for the export):

TS=("total hip arthroplasty" OR "total hip replacement" OR "hip arthroplasty")
AND TS=(frail* OR "frailty index" OR "modified frailty index" OR "mFI-5" OR "mFI-11")
AND TS=("older adult" OR "older adults" OR elderly OR geriatric* OR aged)
AND TS=(
 "intensive care unit" OR "intensive care units" OR ICU OR "critical care"
 OR outcome* OR prognos* OR mortality OR complication*
 OR readmission* OR "length of stay"
)

## Merge and deduplication record

The two original exports comprised 468 records (235 PubMed plus 233 Web of Science). Conservative cross-database deduplication removed 106 duplicate records and retained 362 unique records for subsequent relevance screening.

# Targeted supplementary PubMed search

Search date: 29 July 2026

(("Arthroplasty, Replacement, Hip"[Mesh] OR "hip arthroplast*"[Title/Abstract] OR "hip replacement*"[Title/Abstract] OR hemiarthroplast*[Title/Abstract] OR "revision hip"[Title/Abstract]) AND ("Clinical Frailty Scale"[Title/Abstract] OR "Hospital Frailty Risk Score"[Title/Abstract] OR "Risk Analysis Index"[Title/Abstract] OR sarcopenia[Title/Abstract] OR "comprehensive geriatric assessment"[Title/Abstract] OR "physical frailty"[Title/Abstract] OR frailty[Title/Abstract])) AND ("1900/01/01"[Date - Publication] : "2026/07/29"[Date - Publication])

The supplementary search retrieved 292 records. Of these, 151 matched the original 468-record export and 141 were additional records. Title-and-abstract screening identified 34 high-priority additional records. Six direct or higher-level records were incorporated into the manuscript.

# Eligibility, relevance screening, and selection for narrative synthesis

Records were eligible when they addressed a frailty measure or a defined physical, nutritional, cognitive, or social-functional vulnerability in elective primary THA, revision THA, fracture-related THA/hemiarthroplasty, or a mixed-joint/hip-fracture population needed to address an evidence gap. Duplicates, non-human studies, records without relevant vulnerability or outcome content, and records unable to support a substantive statement were excluded. Grey literature, registries, conference abstracts, and non-peer-reviewed reports were not used as primary evidence. Screening was based on title and abstract and was designed for a framework-oriented narrative synthesis rather than a systematic-effect review.

For the 29 July 2026 supplementary search, the archived script assigned 6 points when the title contained total hip arthroplasty, hip arthroplasty, hip replacement, or hemiarthroplasty; 5 points when the title contained frailty, frail, or sarcopenia; 4 points when the title or abstract contained Clinical Frailty Scale, Hospital Frailty Risk Score, Risk Analysis Index, or comprehensive geriatric assessment; 2 points for revision, fracture, functional, mortality, complication, discharge, delirium, or intensive care content; and 2 points for a review, meta-analysis, or guideline publication type. Among 141 records not matched to the original export, 34 scored at least 9 and were labelled high priority for manual scientific review. The threshold was a reproducible relevance triage rule, not a risk-of-bias or certainty assessment. Six records supplied direct or higher-level evidence used in the text; the other high-priority records informed mapping or did not support a distinct final claim.

# Narrative evidence-selection flow

Supplementary Figure S1. Narrative evidence identification and selection. Counts document the original and supplementary searches, deduplication, relevance screening, and sources cited in the revised manuscript. The diagram is provided for transparency and is not presented as a PRISMA systematic-review flow. One reviewer-suggested source was added during revision outside the dated searches.

# Sources incorporated after the targeted supplementary search

Six records identified through the project-specific relevance-triage procedure were incorporated into the narrative synthesis after manual scientific review. The triage procedure was used only to prioritize records for review and did not assess methodological quality, risk of bias, or certainty of evidence.

| **PMID** | **Year** | **Title** | **DOI** |
| --- | --- | --- | --- |
| 38901711 | 2024 | Prevalence and Clinical Impact of Sarcopenia in Patients Undergoing Total Joint Arthroplasty: A Systematic Review and a Meta-Analysis. | 10.1016/j.arth.2024.06.021 |
| 40025603 | 2025 | Clinical frailty scale predicts outcomes following total joint arthroplasty. | 10.1186/s42836-024-00294-8 |
| 40212940 | 2025 | The risk analysis index predicts 30-day periprosthetic fracture following primary total hip arthroplasty. | 10.1016/j.jor.2025.03.018 |
| 42355658 | 2026 | The Prognostic Value of Frailty Assessment Tools in Predicting Postoperative Outcomes After Revision Total Hip and Knee Arthroplasty: A Systematic Review. | 10.3390/jcm15124489 |
| 42226691 | 2026 | Frailty Assessment in Total Hip Arthroplasty for Femoral Neck Fracture. | 10.5371/hp.2026.38.2.180 |
| 35999509 | 2022 | The association between frailty and mortality among lower limb arthroplasty patients: a systematic review and meta-analysis. | 10.1186/s12877-022-03369-w |

**Reviewer-suggested source incorporated during revisio**

Karahan M, Ozdemir E, Kina S. Integrated nutritional-inflammatory and frailty-based model for mortality risk stratification following hip fracture surgery: a multicentre cohort study. Aging Clin Exp Res. 2026;38:95. <https://doi.org/10.1007/s40520-026-03345-z>. This study appears as reference 49 in the revised manuscript. Because the cohort comprised hip-fracture surgery patients without a separately reported arthroplasty subgroup, it was treated as indirect evidence for arthroplasty-specific questions.
